# Identification of a Tissue-Selective Heat Shock Response Regulatory Network

**DOI:** 10.1371/journal.pgen.1003466

**Published:** 2013-04-18

**Authors:** Eric Guisbert, Daniel M. Czyz, Klaus Richter, Patrick D. McMullen, Richard I. Morimoto

**Affiliations:** 1 Department of Molecular Biosciences, Rice Institute for Biomedical Research, Northwestern University, Evanston, Illinois, United States of America; 2 Department of Chemical and Biological Engineering, Northwestern University, Evanston, Illinois, United States of America; The University of Texas Health Science Center at Houston, United States of America

## Abstract

The heat shock response (HSR) is essential to survive acute proteotoxic stress and has been studied extensively in unicellular organisms and tissue culture cells, but to a lesser extent in intact metazoan animals. To identify the regulatory pathways that control the HSR in *Caenorhabditis elegans*, we performed a genome-wide RNAi screen and identified 59 genes corresponding to 7 positive activators required for the HSR and 52 negative regulators whose knockdown leads to constitutive activation of the HSR. These modifiers function in specific steps of gene expression, protein synthesis, protein folding, trafficking, and protein clearance, and comprise the metazoan heat shock regulatory network (HSN). Whereas the positive regulators function in all tissues of *C. elegans*, nearly all of the negative regulators exhibited tissue-selective effects. Knockdown of the subunits of the proteasome strongly induces HS reporter expression only in the intestine and spermatheca but not in muscle cells, while knockdown of subunits of the TRiC/CCT chaperonin induces HS reporter expression only in muscle cells. Yet, both the proteasome and TRiC/CCT chaperonin are ubiquitously expressed and are required for clearance and folding in all tissues. We propose that the HSN identifies a key subset of the proteostasis machinery that regulates the HSR according to the unique functional requirements of each tissue.

## Author Summary

The heat shock response (HSR) is an essential stress response that functions to maintain protein folding homeostasis, or proteostasis, and whose critical role in human diseases is recently becoming apparent. Previously, most of our understanding of the HSR has come from cultured cells and unicellular organisms. Here we present the identification of the heat shock regulatory network (HSN) in *Caenorhabditis elegans*, an intact, multicellular organism, using genome-wide RNAi screening. We identify 59 positive and negative regulators of the HSR, all of which have a previously established role in proteostasis, linking the function of the HSR to its regulation. Some HSN genes were previously established in other systems, many were indirectly linked to HSR, and others are novel. Unexpectedly, almost all negative regulators of the HSR act in distinct, tissue-selective patterns, despite their broad expression and universal cellular requirements. Therefore, our data indicate that the HSN consists of a specific subset of the proteostasis machinery that functions to link the proteostasis network to HSR regulation in a tissue-selective manner.

## Introduction

The heat shock response (HSR) has been studied extensively as a cellular response to acute stress such as elevated temperature [1]. The master regulator of the HSR is Heat Shock Factor 1 (HSF1), a stress responsive transcription factor that regulates the inducible transcription of a family of genes encoding heat shock proteins (HSPs), many of which are molecular chaperones. In the absence of a stress signal, HSF1 is inhibited by a negative feedback loop mediated by the molecular chaperones HSP70 and HSP90 [2]–[7]. Upon heat shock, HSF1 is activated as the equilibrium of chaperones shifts toward association with metastable polypeptides.

Many key aspects of the HSR have been well established at a cellular level in cultured cells and unicellular organisms, yet the HSR has additional features that are only apparent in multicellular organisms. Heat shock inducible promoters contain multiple *cis* elements and can be differentially expressed across tissues [8]–[18]. The HSR is intimately associated with numerous tissue-specific and age-dependent human diseases and regulated cell non-autonomously by neuronal control [19], [20]. Finally, HSF1 has important roles during development and longevity, and activation of the HSR is attenuated during aging [13], [21]–[24]. However, despite the importance of the HSR in organismal physiology, relatively little is known about its regulation in multicellular organisms and the extent of differential regulation across distinct tissues is unexplored.

A comprehensive genetic analysis of the HSR regulatory pathways has not previously been possible in any system, in part because traditional forward genetic screens are inadequately suited to the identification of genes that regulate the HSR. These approaches depend on the introduction of mutations, which can destabilize the folding of the corresponding proteins, resulting in indirect induction of the HSR due to the expression of misfolded species. Indeed, a forward genetic screen in *Drosophila* described such mutations in a muscle-specific actin [25], [26]. RNAi based genetic screening resolves the limitations associated with traditional genetic screens associated with the HSR and has been used to gain important insights into many regulatory networks including those associated with models of aggregation-prone proteins, longevity, and stress responses [27]–[34].

In this study, we have used genome-wide RNAi screening to identify factors important for the positive and negative regulation of the HSR in the metazoan *Caenorhabditis elegans* in order to establish a comprehensive understanding of its regulation on an organismal level. Further, we used a fluorescent reporter to allow for the analysis of regulation in different tissues. This approach reveals a complex network of positive and negative HSR regulators with critical roles in maintenance of proteostasis that confer differential tissue-selective patterns of heat shock gene expression.

## Results

### Genome-Wide Screens for HSR Regulators

**Figure 1:**
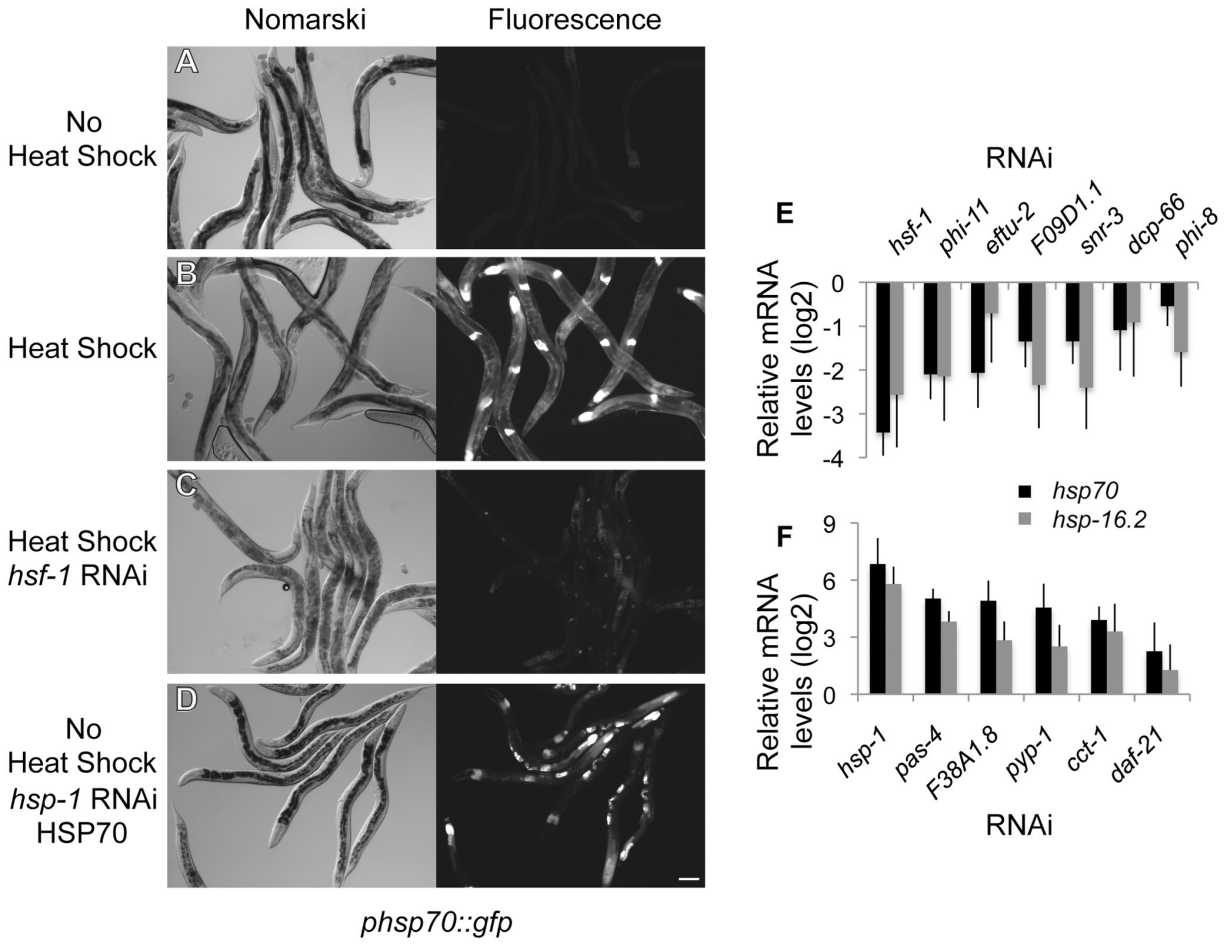
Genome-wide RNAi screen for HSR regulators. (A–D) Nomarski and fluorescent images corresponding to the *phsp70::gfp* reporter strain. (A) Control animals show little reporter expression and only faint autofluorescence of the intestine. (B) Reporter induction in animals exposed to heat shock at 33°C for 1 hour. (C) RNAi knockdown of *hsf-1* decreases induction of the reporter by heat shock. (D) RNAi knockdown of *hsp-1* causes constitutive induction of the reporter in the absence of heat shock. The scale bar corresponds to 100 µm. (E–F) Quantitation of the effects on endogenous *hsp70* and *hsp-16.2* genes using qRT-PCR. (E) HSR positive regulators normalized to the heat shocked empty vector control. (F) HSR negative regulators normalized to empty vector control. Averages are from at least three biological replicates and error bars represent SEM.

The genetic network upstream of HSF1 and the HSR was identified using a genome-wide RNAi screen in transgenic *C. elegans* expressing the heat shock (HS)-inducible fluorescent reporter *phsp70::gfp* constructed from the promoter of the *C12C8.1* gene [13]. Expression of this reporter is not detected under ambient growth conditions of development and adulthood (Figure 1A) and is induced strongly by HS (Figure 1B). The threshold sensitivity of the screen was established using RNAi knockdown of *hsf-1* to suppress HS-induction of the reporter as a reference control for positive regulators (Figure 1C), and RNAi knockdown of *hsp-1*, a member of the HSP70 family that negatively regulates the HSR, resulting in constitutive expression of the reporter as a reference control for negative regulators (Figure 1D).

Genetic modifiers of the HSR were identified by visual scoring of the *phsp70::gfp* reporter upon RNAi-mediated knockdown. A representative subset from each functional class was validated by analysis of endogenous *hsp70* gene expression using qRT-PCR (Figure 1E and 1F). We also extended our analysis to another heat shock gene, *hsp-16.2*, a member of the small HSP family. Consistent with the HSR reporter results, HS-dependent induction of *hsp70* and *hsp-16.2* were reduced upon *hsf-1* knockdown. Likewise, the basal expression of *hsp70* and *hsp-16.2* were increased upon *hsp-1* knockdown. These experiments established the utility of the *phsp70::gfp* reporter and RNAi as a methodology for the identification of both positive and negative regulators of the HSR in *C. elegans*.

Having established the criteria for two classes of HSR genetic modifiers, we performed a genome-wide RNAi screen for genes whose knockdown blocked HS-dependent reporter induction, and for genes whose knockdown resulted in constitutive expression of the reporter. These screens were performed by RNAi feeding using a library containing RNAi constructs targeted against approximately 86% of genes in *C. elegans* [35].

### Identification of HSR Positive Regulators

The screen for positive regulators of the HSR identified genes with properties similar to *hsf-1*, whose knockdown suppressed induction of the HSR reporter. To ensure that decreased fluorescence of the reporter did not arise from indirect effects, such as transgene silencing, we performed a counter-screen against suppression of a *phsp-4::gfp* reporter, an ER stress-inducible gene that is not dependent on HSF1 [36], [37]. This led to the identification of seven positive regulators that are conserved to humans and function in chromatin remodeling, RNA processing, and protein synthesis (Figure 1E, Table 1, Table S1). None of these genes has been previously linked to HSR regulation, however each has been either associated with HS or implicated in the HSR. For example, *dcp-66* is a subunit of the NuRD complex, of which other subunits in this complex have been shown to interact with human HSF1 [38]. Our data provide evidence that the HSF1-NuRD interaction has functional consequences on the regulation of the HSR. Likewise, Mi-2, a subunit of several complexes including NuRD, has been shown to affect the levels of HS genes in *Drosophila* [39]. Among the other positive regulators are genes associated with mRNA splicing and translation, biosynthetic processes that are highly sensitive to HS stress. *F09D1.1* is a homologue of USP39, which has been implicated in recycling of the triple-snRNP complex, a step of splicing that is particularly sensitive to temperature. *phi-8* and *phi-11* are subunits of Splicing Factor 3, which has been shown to regulate alternative splicing, *snr-3* is an sm protein which is expected to have a general role in mRNA splicing, and *eftu-2* is an elongation factor 2-like protein predicted to have a general role in translational elongation. Finally, as expected, *hsf-1* was identified in the screen as predicted for its central role in the HSR.

**Table 1:** Positive Regulators of the HSR.

Cosmid	Gene	Description
ZK328.2	*eftu-2*	EF-2 like
C26C6.5	*dcp-66*	NuRD subunit
F09D1.1	*F09D1.1*	USP39
T08A11.2	*phi-11*	Splicing Factor 3B, subunit 1
T13H5.4	*phi-8*	Splicing Factor 3A, subunit 3
T28D9.10	*snr-3*	SNRPD1
Y53C10A.12	*hsf-1*	Heat Shock Factor

### Identification of HSR Negative Regulators

The screen for negative regulators of the HSR identified genes whose reduced expression resulted in the constitutive expression of the *phsp70::gfp* reporter. To ensure that these regulators activated the HSR in an HSF1-dependent manner, we employed a subsequent counter-screen using a hypomorphic *hsf-1* mutant [40]. Candidate negative regulators were also tested for their ability to constitutively activate endogenous heat shock genes by qRT-PCR (Figure 1F). This strategy led to the identification of fifty-two genes that have the functional properties of negative regulators of the HSR (Table 2, Table S1).

**Table 2:** Negative regulators of the HSR and *hsp70::gfp* reporter induction.

Cosmid	Gene	Description	S	I	M
F26D10.3	*hsp-1*	HSP70 Chaperone	•	•	•
C47E8.5	*daf-21*	HSP90 Chaperone	•	•	•
T05C12.7	*cct-1*	CCT/TRiC Chaperone	○	○	•
T21B10.7	*cct-2*	CCT/TRiC Chaperone	○	○	•
F54A3.3	*cct-3*	CCT/TRiC Chaperone	○	○	•
K01C8.10	*cct-4*	CCT/TRiC Chaperone	○	○	•
C07G2.3	*cct-5*	CCT/TRiC Chaperone	○	○	•
F01F1.8	*cct-6*	CCT/TRiC Chaperone	○	○	•
T10B5.5	*cct-7*	CCT/TRiC Chaperone	○	○	•
Y55F3AR.3	*cct-8*	CCT/TRiC Chaperone	○	○	•
R05F9.10	*sgt-1*	TPR cochaperone	○	○	•
F30H5.1	*unc-45*	TPR cochaperone	○	○	•
T01B7.4	*cyn-11*	Cyclophilin cochaperone	○	○	•
C36B1.4	*pas-4*	Proteasome 20S subunit	•	•	○
F25H2.9	*pas-5*	Proteasome 20S subunit	•	•	○
CD4.6	*pas-6*	Proteasome 20S subunit	•	•	○
C47B2.4	*pbs-2*	Proteasome 20S subunit	•	•	○
Y38A8.2	*pbs-3*	Proteasome 20S subunit	•	•	○
T20F5.2	*pbs-4*	Proteasome 20S subunit	•	•	○
K05C4.1	*pbs-5*	Proteasome 20S subunit	•	•	○
C02F5.9	*pbs-6*	Proteasome 20S subunit	•	•	○
F39H11.5	*pbs-7*	Proteasome 20S subunit	•	•	○
C52E4.4	*rpt-1*	Proteasome 19S subunit	•	•	○
F23F12.6	*rpt-3*	Proteasome 19S subunit	•	•	○
F23F1.8	*rpt-4*	Proteasome 19S subunit	•	•	○
F56H1.4	*rpt-5*	Proteasome 19S subunit	•	•	○
Y49E10.1	*rpt-6*	Proteasome 19S subunit	•	•	○
T22D1.9	*rpn-1*	Proteasome 19S subunit	•	•	○
C23G10.4	*rpn-2*	Proteasome 19S subunit	•	•	○
F57B9.10	*rpn-6*	Proteasome 19S subunit	•	•	○
F49C12.8	*rpn-7*	Proteasome 19S subunit	•	•	○
R12E2.3	*rpn-8*	Proteasome 19S subunit	•	•	○
K07D4.3	*rpn-11*	Proteasome 19S subunit	•	•	○
F57B10.1	*let-607*	Transcription Factor (ER)	•	•	○
C15H9.6	*hsp-3*	HSP70 Chaperone (ER)	•	•	○
F38A1.8		SRP receptor α subunit	•	•	○
R186.3		SRP receptor β subunit	•	•	○
F55C5.8		SRP subunit	•	•	○
F08D12.1		SRP subunit	○	•	○
F25G6.8		SRP subunit	○	•	○
F38E11.5		COPI β' subunit	○	•	○
T14G10.5		COPI γ subunit	○	•	○
T24H7.2		HSP70 Chaperone (ER)	○	•	○
C37H5.8	*hsp-6*	HSP70 Chaperone (mito)	○	•	○
T09B4.9		TIM44 subunit (mito)	○	•	○
C47E12.5	*uba-1*	E1 ubiquitin ligase	○	•	○
F52C6.3	*phi-32*	Ubiquitin	○	•	○
C53A5.6		E3 ubiquitin ligase	○	•	○
B0464.1	*dars-1*	Asp tRNA Synthetase	○	•	○
W04A4.5		Integrator subunit	○	•	○
C47E12.4	*pyp-1*	NuRF subunit	○	•	○
R12B2.5	*mdt-15*	Mediator subunit	○	•	○

S = Spermatheca, I = Intestine, M = Muscle, • = Induction, ○ = No Induction.

Each of these negative regulators of the HSR function in specific steps of proteostasis and affect either gene expression, protein folding, trafficking, and clearance, and are conserved to humans. Among the regulators that affect protein folding are three prominent molecular chaperone machines corresponding to HSP70 (*hsp-1*), HSP90 (*daf-21*) and TRiC/CCT (*cct-1, cct-2, cct-3, cct-4, cct-5, cct-6, cct-7, and cct-8*) and three cochaperones (*sgt-1*, *unc-45*, and *cyn-11*). HSP70 and HSP90 are predicted from previous studies that identified them as negative regulators of HSF1 and the HSR. Likewise, a role for the TRiC/CCT chaperonin in the regulation of the HSR has been suggested from studies on a small molecule that interacts with TRiC/CCT and induces human HSF1 [41]. Regulation of the HSR by chaperonins is functionally conserved in bacteria, as downregulation of the prokaryotic chaperonin GroEL induces the HSR in *E. coli* [41], [42]. The selectivity of these genes representing three chaperone machines and three cochaperones as regulators of the HSN is unexpected given that *C. elegans* expresses nearly 200 chaperone genes, which suggests a high degree of selectivity for chaperone regulation of the HSR.

Other negative regulators of the HSR correspond to components of trafficking including subunits of the signal recognition particle (SRP) and other secretory pathway genes (*F55C5.8*, *F08D12.1*, *F25G6.8*, *F38A1.8*, *R186.3*, *F38E11.5* and *T14G10.5*, *hsp-3*, *T24H7.2*, *let-607*) and mitochondrial import (*hsp-6*, *T09B4.9*). Consistent with this, knockdown of SRP subunits in yeast and *E. coli* has been shown to induce the HSR [43], [44] and our study now extends these observations to metazoans. Clearance components include ubiquitin associated (*phi-32*, *uba-1*, *C53A5.6*) and proteasomal subunits (*pas-4*, *pas-5*, *pas-6*, *pbs-2*, *pbs-3*, *pbs-4*, *pbs-5*, *pbs-6*, *pbs-*7, rpt*-1*, *rpt-3*, *rpt-4*, *rpt-5*, *rpt-6*, *rpn-1*, *rpn-2*, *rpn-6*, *rpn-7*, *rpn-8*, and *rpn-11*). Inhibition of the proteasome by small molecules has previously been shown to induce the HSR [45], [46]. It is intriguing that only the proteasome, and not autophagy or other proteases, functions as a regulator of the HSR, given the large number of components involved in protein clearance. The final class of regulators are involved in protein synthesis (*dars-1*) and gene expression (*W04A4.5*, *pyp-1*, and *mdt-15*). Microarray results confirm the induction of HSR genes upon *mdt-15* knockdown [47]. While the *pyp*-1 subunit of the NuRF chromatin remodeler has not been previously linked to the HSR, other subunits of NuRF have been suggested to positively affect HSR gene expression [48]. Because we identified only one of 171 predicted E3-ligases (*C53A5.6*) and only one of 33 predicted tRNA synthetases (*dars-1*) in the *C. elegans* genome, we rescreened all members of these gene families and found no additional HSR regulators [49], [50].

### HSR Negative Regulators Exhibit Distinct Tissue-Selective Patterns

**Figure 2:**
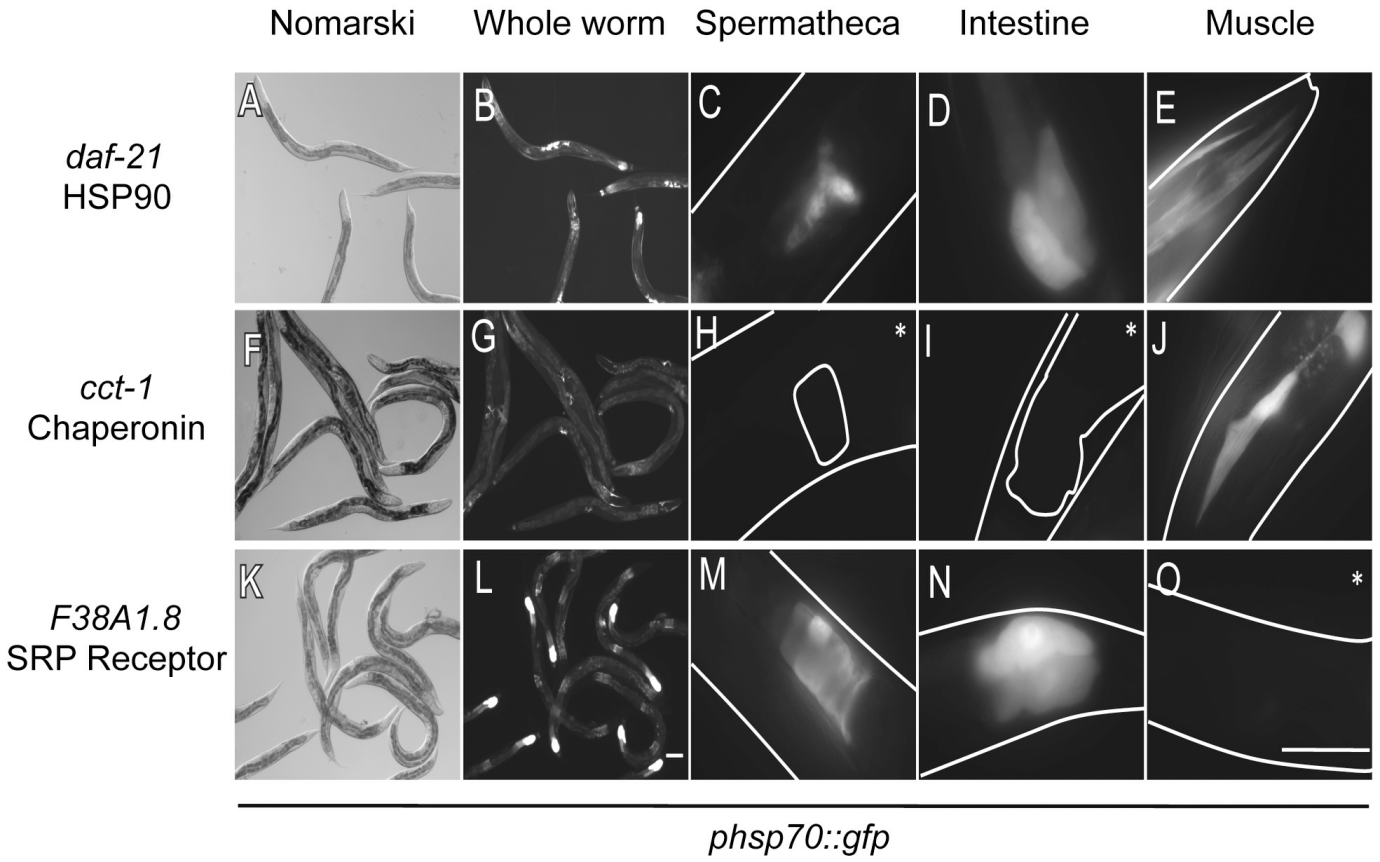
Tissue-selective induction of the ***phsp70::gfp*** **reporter by knockdown of negative regulators.** Nomarski and fluorescent images corresponding to whole animals and fluorescent images of the spermatheca, intestine, and muscle tissue are shown. The boundary of the animals, intestine and spermatheca taken from Nomarski images are added as a visual aide to some images. (A–E) RNAi knockdown of *daf-21* leads to induction of the reporter in all three tissues. (F–J) RNAi knockdown of *cct-1* causes induction only in muscle. (K–O) knockdown of *F38A1.8* causes induction in the intestine and spermatheca. Images are taken at different exposures to maximize fluorescence of each image. Scale bars of whole animal images correspond to 100 µm, while scale bars of the images depicting specific tissues correspond to 50 µm. Asterisks denote only autofluorescence.

A striking feature of the negative regulators of the HSR is that the HSR reporter in not uniformly induced across all tissues, but rather displays tissue-selective expression patterns in the intestine, muscle, and spermatheca (Figure 2, Table 2). Of the negative regulators, only knockdown of HSP70 and HSP90 induced expression of the reporter in all three tissues. By comparison, knockdown of the three cochaperones and all eight subunits of the TRiC/CCT chaperone machine induced the reporter only in the muscle. In contrast, downregulation of subunits of the proteasome and many secretory pathway genes induced the reporter only in the intestine and spermatheca, but not in the muscle. Knockdown of the remaining genes induced the reporter only in the intestine. These patterns are unlikely to be due to RNAi artifacts because the tissue-selective patterns of reporter induction were similar for all subunits within specific complexes, yet non-overlapping between different complexes (i.e., all proteasomal subunits induced in intestine and spermatheca, and all TRiC/CCT subunits induced in the muscle).

Tissue-selective expression of the HSR reporter was unexpected as nearly all of the negative regulators are ubiquitously expressed components of essential cellular machines. For example, even though proteasomal subunits do not induce the HSR in muscle, it has been shown in *C. elegans* that most, if not all, proteasomal subunits are expressed in muscle and that RNAi knockdown of proteasomal subunits yields muscle specific phenotypes such as stabilization of a ubiquitin-GFP reporter in the muscle and early onset aggregation of a polyQ disease model expressed only in the muscle [28], [51]. These results also suggest that depletion of subunits from complexes such as the proteasome does not induce the HSR simply by misfolding other subunits in that complex since these effects would not be expected to have tissue selectivity.

To further investigate the tissue-selective patterns of HSR regulation, we examined the expression of two additional reporters that are inducible by heat shock and dependent on HSF1. The *phsp-16.2::gfp* reporter is inducible in the intestine, muscle and excretory system, and is dependent on HSF1 and DAF-16 (Figure S1) [52], [53]. The *pckb-2::gfp* reporter is inducible only in the intestine and is also activated by the unfolded protein response, an ER stress response [54]. Knockdown of HSR negative regulators revealed highly overlapping patterns of tissue-specific induction with all three reporters (Table 3). In the muscle, there was a highly consistent pattern of induction between the *C12C8.1* and *hsp16.2* reporters, with two genes inducing both, seven inducing neither, and only a single gene showing differential induction. In the intestine, HSR negative regulators gave identical patterns of induction for the *C12C8.1* and the *ckb-2* reporters, with nine out of ten causing induction, yet only a smaller subset, three out of ten, also induced the *hsp16.2* reporter. Given the differences in the regulation and function of the three genes, the three reporters demonstrate remarkably consistent patterns of tissue-selective HSR induction.

**Table 3:** Tissue-selective patterns for multiple HSR reporters.

		*hsp70*		*ckb-2*	*hsp16.2*	
Gene	Description	I	M	I	I	M
*hsp-1*	HSP70	•	•	•	•	○
*daf-21*	HSP90	•	•	•	○	•
*cct-1*	CCT/TRiC	○	•	○	○	•
*pas-4*	Proteasome	•	○	•	○	○
*let-607*	Transcription Factor	•	○	•	○	○
*F38A1.8*	SRP receptor α	•	○	•	•	○
*hsp-6*	HSP70 (mito)	•	○	•	○	○
*C53A5.6*	E3 ubiquitin ligase	•	○	•	○	○
*dars-1*	tRNA Synthetase	•	○	•	○	○
*pyp-1*	NuRF	•	○	•	•	○

I = Intestine, M = Muscle, • = Induction, ○ = No Induction.

**Figure 3:**
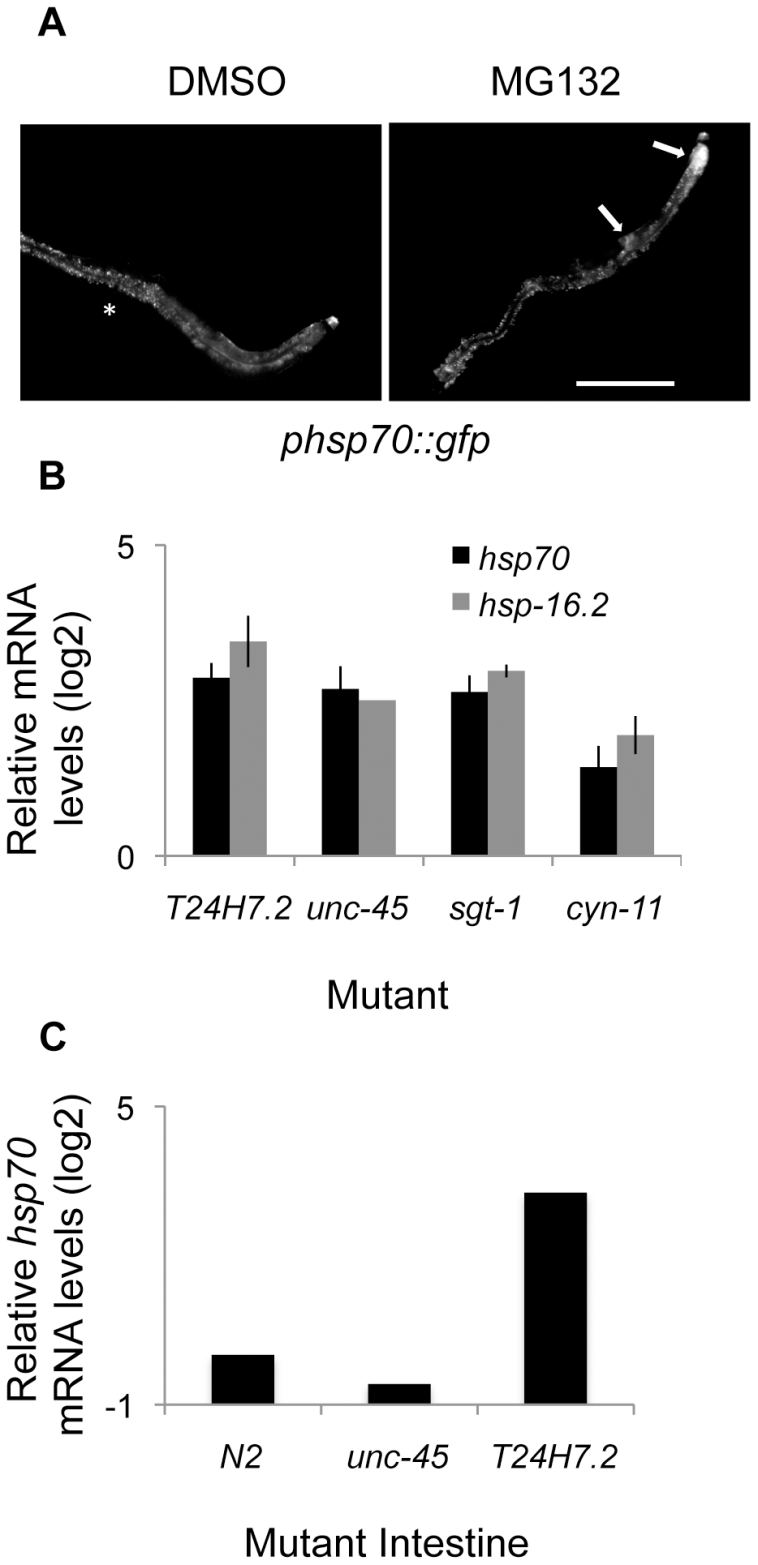
Validation of tissue-selective effects using small molecules and mutants. A) Incubation with 100 µM MG132, a small molecule inhibitor of the proteasome, but not DMSO alone, causes tissue-selective induction of the *phsp70::gfp* reporter in the intestine and spermatheca (arrows), similar to RNAi knockdown of proteasomal subunits. The scale bar corresponds to 200 µm. Asterisks denote only autofluorescence. B) Mutations in *T24H7.2, sgt-1, cyn-11, and unc-45* cause induction of the HSR in whole worms measured using qRT-PCR. C) Mutation of *T24H7.2*, but not *unc-45*, causes induction in the intestine relative to N2 control animals, measured by qRT-PCR analysis of *hsp70* in dissected intestinal tissue. Averages shown are from at least two biological replicates.

We next validated the tissue-selective effects using pharmacological inhibitors and mutants. We found that incubation of L4-staged worms with MG132, a pharmacological inhibitor of the proteasome, caused induction of the *phsp70::gfp* reporter in the intestine and the spermatheca, but not in the muscle tissue (Figure 3A). This pattern matches that seen with RNAi knockdown of proteasomal subunits, providing further support that the tissue-selective effects are unlikely to be an RNAi artifact. Most of the negative regulators are essential, however we were able to test the effects of mutations in *T24H7.2*, an ER localized HSP70, and the cochaperones *unc-45*, *sgt-1*, and *cyn-11*, and found using qRT-PCR that the expression of endogenous HSR genes was induced (Figure 3B). We further demonstrated that this induction was tissue-selective using qRT-PCR analysis on dissected intestinal cells. We found that *T24H7.2* mutant animals, but not *unc-45* mutant animals, induced endogenous *hsp70* in the intestine (Figure 3C). The tissue-selective induction of endogenous genes in the intestine by these mutations matched the induction of the *phsp70::gfp* reporter by RNAi knockdown, thus providing a validation of both the use of RNAi and the fluorescent reporter.

### Analysis of the HSR Regulatory Network

**Figure 4:**
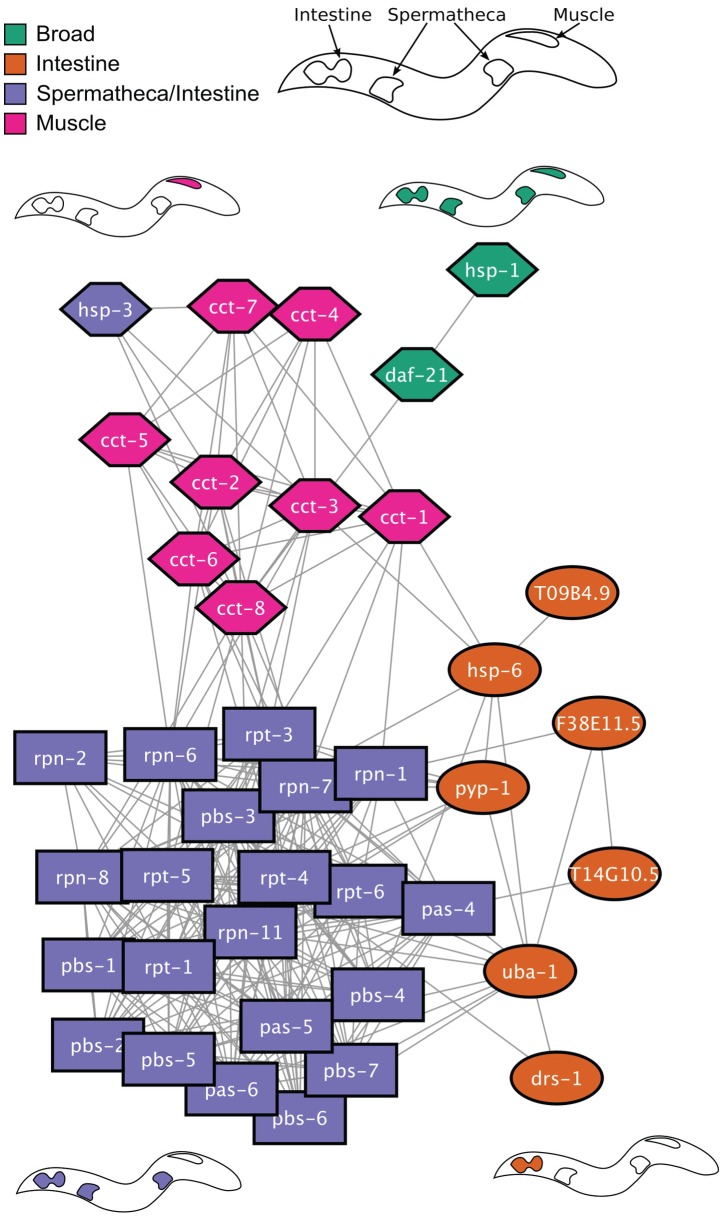
Network analysis of HSR regulators. Shown is a network with HSR negative regulator genes depicted as nodes and interactions as edges. Node shape denotes grouping corresponding to a community detection algorithm based on the structure of the interaction network. Node color corresponds to the tissue-specific *phsp70::gfp* reporter induction. Cartoons of worms depicting the tissue specificity appear next to nodes containing those colors.

The genes that we identified form a genetic regulatory network of the HSR in *C. elegans*. To characterize the relationship between these regulators, we utilized an interaction network from previous physical, genetic, and predicted interaction data [55]. A network representation of the interaction data, in which HSR regulators are nodes and interactions between them are edges, reveals that HSR negative regulators are enriched in interactions with other HSR negative regulators: 39 of 52 negative regulator genes are connected in a single interaction network (Figure 4). We next applied a community detection algorithm to determine the structure of this interaction network [56], [57]. This analysis shows that the network is composed of three distinct modules, indicated by the shapes of the nodes. The modular structure of this network is unlikely to have arisen by chance since it does not appear in randomized networks containing the same number of nodes and connections (p<10^−4^). The three modules are primarily composed of clearance, cytoplasmic protein folding, and gene expression and protein synthesis components, respectively. While it is unsurprising that proteasome or protein folding subunits cluster together into distinct modules, the existence of the third module is entirely unexpected. The modules identified using the interaction data (node shapes) correspond closely with the observed tissue patterns of reporter induction (node colors) thus providing additional validation of both the specificity of tissue expression and network structure. These results further suggest that the underlying functional modules give rise to the tissue-specific patterns of HSR induction.

**Figure 5:**
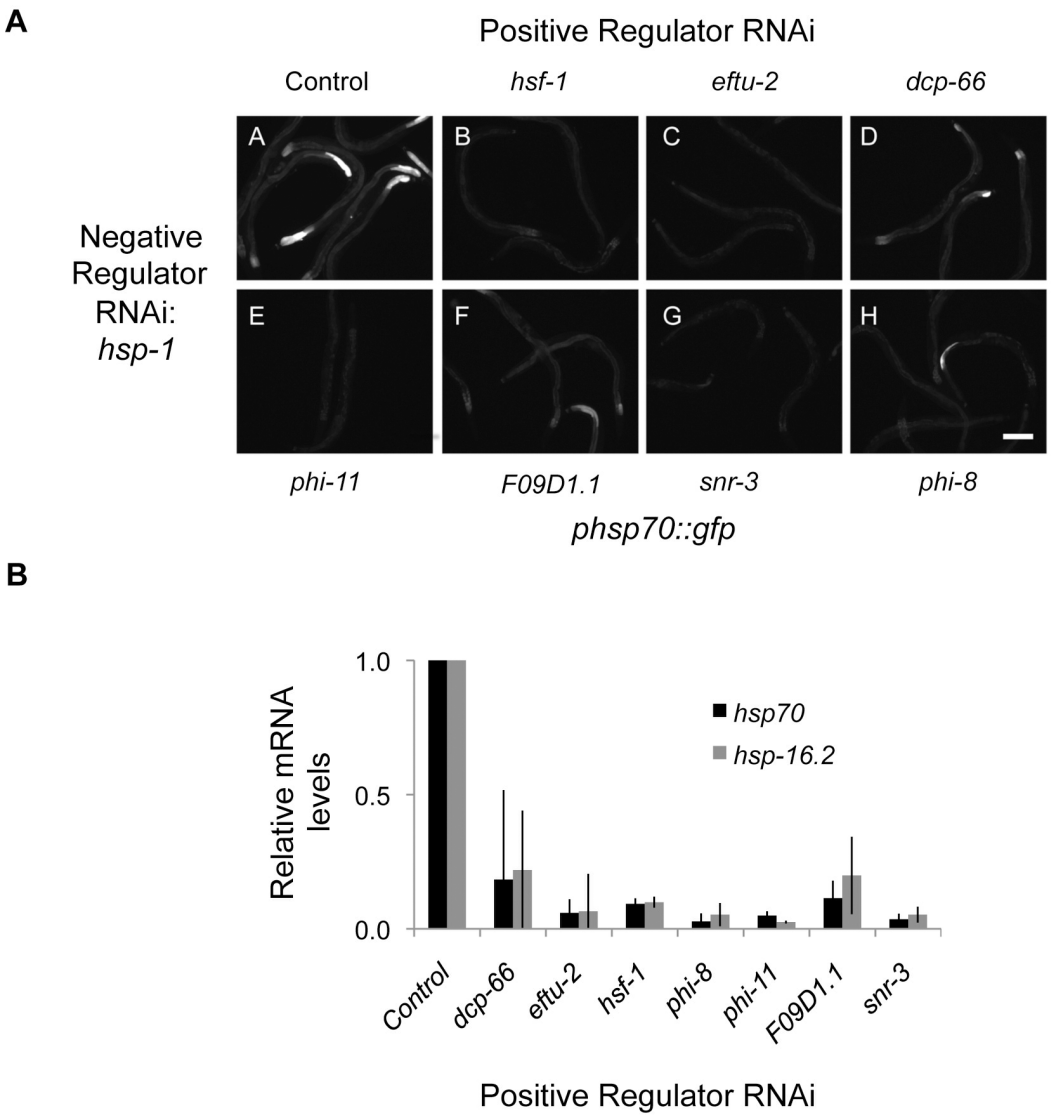
Epistasis analysis of HSR regulators. The effects of HSR positive regulator knockdown on induction of the reporter by negative HSR regulator knockdown were measured using the *phsp70::gfp* reporter. (A) Images showing the results from double RNAi with each positive regulator and the negative regulator *hsp-1*. In each case, knockdown of the positive regulator decreased reporter fluorescence compared to knockdown of *hsp-1* alone. (B) Quantitation of the effects of HSR positive regulator knockdown using RNAi on induction of endogenous HSR genes by the HSR negative regulator *T24H7.2* mutant reveals that the positive regulators are epistatic to *T24H7.2*.

To further probe the genetic properties of the HSN, we investigated the relationships between the positive and negative regulators to provide a systems-level pathway analysis. We tested whether depletion of positive regulators (that decrease reporter induction by heat shock) would suppress reporter induction mediated by depletion of negative regulators. We found that knockdown of each positive regulator prior to knockdown of the negative regulator *hsp-1* (HSP70) decreased induction of the reporter (Figure 5A). This indicates that the positive HSR regulators are epistatic to HSP70. These data are consistent with a model in which the positive regulators of the HSR act at or downstream of chaperone-mediated regulation of the HSR. Similar results were obtained for other negative regulators including *daf-21* (HSP90), *pas-4* (proteasome), *C53A5.6* (E3 ubiquitin ligase), *let-607* (ER transcription factor), *F38A1.8* (SRP), *hsp-6* (mitochondrial HSP70), and *dars-1* (Asp tRNA synthetases).

**Figure 6:**
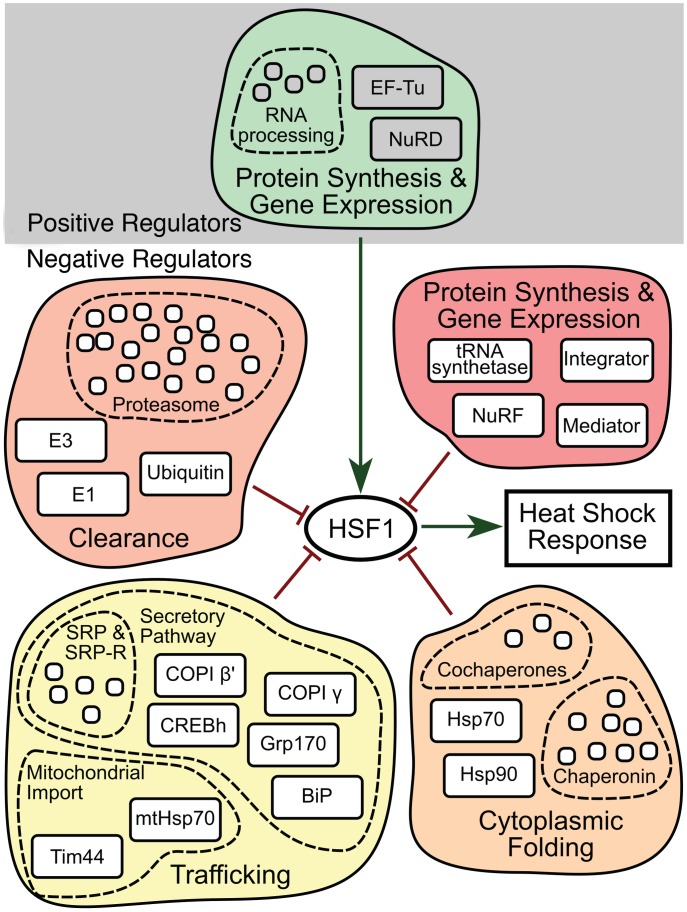
HSR regulatory network model. Each HSR regulator, denoted by common terminology, is indicated as a box and is grouped according to its presence in a multi-subunit complex or functional pathway (i.e., the proteasome or secretory pathway). Positive or negative effects on HSR regulation are indicated by either a green arrow or red line respectively. Positive regulators are further separated from negative regulators by grey shading in the background. At the center of the network, HSF1 integrates signals from the various regulators and establishes a coordinated HSR.

These results, in addition to the tissue-independent nature of the positive regulators and their association with biosynthetic processes, further distinguish the roles of the positive regulators from the negative regulators. RNAi knockdown is not equivalent to genetic ablation, so these relationships correspond to sensitivities rather than absolute dependencies. Therefore, we tested the effects of depletion of the positive regulators in a strain containing a deletion in the negative regulator *T24H7*.2, an ER localized HSP70. Each of the positive regulators decreased induction of the HSR upon mutation of *T24H7*.2, thereby confirming the results with double RNAi (Figure 5B). Together, our data indicate that the positive regulators are epistatic to the negative regulators and either function downstream or at the same step in the pathway. We favor the latter model and propose that the positive and negative regulators function together in an integrated HSR regulatory network (Figure 6).

## Discussion

Our findings indicate that the heat shock regulatory network (HSN) enables the HSR to sense and respond to a wide range of disruptions in proteostasis, thus providing a direct link between the function of the HSR and its regulation. The four functional clusters of the HSN each identify a small subset of the entire proteostasis machinery that functions in gene expression and protein synthesis, folding, trafficking, and clearance. The negative regulators fall within each of these functional categories, whereas the positive HSR regulators are more restrictive and cluster only to gene regulation and protein synthesis. Previous studies on the mitochondrial stress response have revealed that depletion of specific subunits of electron transport chain complexes leads to induction of the mitochondrial stress response; however in our screen we did not identify subunits of macromolecular complexes as regulators of the HSR, thus revealing differences in how these two compartments detect and respond to a proteostatic imbalance [58].

The negative regulators of the HSN displayed a surprising extent of tissue-selective effects on HS gene expression, which may arise from differences in the expression levels or activity of the regulators between tissues. One clear example of differential tissue specific expression of HSR regulators occurs in activated B cells, that rely heavily rely upon the secretory pathway and exhibit high expression levels of secretory pathway components (for a review, see [59], [60]). But as described in the results section, nearly all components of the HSN are broadly expressed. For example, TRiC/CCT is required for the folding of actin and tubulin, which are expressed in every cell [61]. However, the specialized function of muscle tissue could necessitate a stronger dependency for actin and myosin, which in turn explains the functional requirement for TRiC/CCT and account for the enhanced sensitivity of muscle to TRiC/CCT depletion. In addition to differential sensitivity to the regulators, our results indicate that each tissue exhibits distinct profiles of HS-inducible genes, which likely arises from tissue-specific factors that influence HS gene inducibility. Together, these data indicate that in addition to its unique proteome and specialized function, each tissue may contain a distinct complement of the proteostasis machinery, a differential sensitivity to disruption of proteostasis networks, and a distinct response to proteostasis disruption.

Induction of the HSR has been shown to be protective in multiple models for diseases of protein conformation; therefore, knockdown of the negative regulators and induction of the HSR would be predicted to suppress protein aggregation. Instead, there is substantial overlap (twenty out of fifty-two genes) between the negative regulators of the HSR and a separate genome-wide screen for enhancement (early onset) of polyQ aggregation in muscle (Table S1). The common gene set includes the TRiC/CCT chaperonin (6), HSP70, mitochondrial HSP70, the proteasome (10), ubiquitin, and the E1 ubiquitin ligase. Therefore, it is likely that knockdown of these genes leads to both a disruption of proteostasis and activation of the HSR. Consistent with this model, there is almost no overlap with another genome-wide screen for suppression of polyQ aggregation in muscle. In contrast, knockdown of positive regulators, which suppresses the HSR, would be predicted to cause early-onset polyQ aggregation, and indeed, five of the seven positive HSR regulators have this phenotype and none suppress polyQ aggregation.

The paradigm for HSR regulation has previously focused on HSF1 and the negative feedback loops consisting of the HSP70 and HSP90 chaperones. Our results reveal that the network that regulates the HSR is much larger and corresponds to at least fifty-nine genes of this newly defined HSN. Many of these genes have been previously linked to HSR regulation in other systems, including prokaryotes, suggesting that this regulatory network is likely conserved through evolution. The precise mechanistic links between many of these genes and the HSR and other components of the HSN remains to be defined, and it will be important to investigate whether the tissue-selective regulation of the HSR is also conserved. Nevertheless, the identification of these genes in a comprehensive genetic screen for HSR regulators not only validates their functional properties but also reinforces the evolutionary conservation of the HSR. In summary, the systems-level identification and characterization of the HSR regulatory network described in this paper provides several important insights into regulation of the HSR during stress and provides a basis for future analysis of HSR regulation during development, ageing, and human disease.

## Materials and Methods

### Nematode Strains and Cultures

Nematodes were handled and analyzed using standard laboratory techniques and cultured at 20°C [62]. Worms were synchronized by bleaching with hypochlorite (NaOCl) and hatching overnight in M9. Where indicated, intestines were dissected from living animals in M9 media. All nematode strains were derived from the N2 Bristol wild-type strain. The following strains were used: 1) AM446 rmIs223[*phsp70::gfp*; pRF4(*rol-6*(su1006))]; 2) SJ4005 zcIs[*phsp-4::gfp*]V; 3) CL2070 dvIs70 [*phsp-16.2::gfp*; *rol-6*(su1006)]; 4) BC14636 *dpy-5*(e907) I; sIs13872[*rCesB0285.9::gfp*+pCeh361]; 5) PS3551 *hsf-1*(sy441)I; 6) AM658 *hsf-1*(sy441)I; rmIs223[*phsp70::gfp*; *rol-6* (su1006)]; 7) RB1694 *T24H7.2*(ok2107) II; 8) RB703 *unc-45*(ok468) III; 9) RB1053 *R05F9.10*(ok1000) II; and 10) VC1372, *rab-21&cyn-11*(ok1879) II [13], [37], [40], [52], [63].

### RNAi

Genome-wide RNAi screening was performed using a bacterial feeding approach with a library targeting approximately 86% of the *C. elegans* genome (MRC Geneservice, Cambridge, U.K.). Bacterial cultures were grown overnight in LB with 5 µg/ml tetracycline and 50 µg/ml ampicillin and induced with 1 mM IPTG for four hours. To avoid L1-stage developmental arrest associated with essential genes, L1 larvae were allowed to develop for 19 hours on plates containing OP50 bacteria prior to exposure to RNAi.

The genome-wide screen was performed in 96-well liquid cultures containing approximately 10 animals, 50 µl M9, 5 µg/ml cholesterol, 5 µg/ml tetracycline, 50 µg/ml ampicillin, 0.4 mM IPTG, 0.1 µg/ml fungizone, and 75 µl of RNAi bacterial suspension and grown at 20°C for 60 hours in a temperature-controlled shaker. For the heat shock screens, the animals were sensitized by exposure for two hours at 24°C, 24 hours before screening for reporter induction. The heat shock conditions are at 31.5°C for two hours followed by 24 hours of recovery at 20°C prior to screening for stress-induced fluorescence. Screening was performed using Leica MZ16-FA fluorescence microscope equipped with a GFP2 filter.

Validation and analysis of the regulators from the primary screen were done using solid RNAi plates containing nematode growth medium (NGM) agar with 5 µg/ml tetracycline, 50 µg/ml ampicillin and 1 mM IPTG and seeded with RNAi bacteria. Synchronized worms grown on OP50 bacteria for 19 hours were incubated on RNAi plates for 48 hours before analysis of induction (negative regulators) or wrapped in parafilm and heat shocked in a water bath at 33°C for 1 hour and then recovered for 24 hours prior to analysis (positive regulators). Worms were immobilized in levamisole and imaged using either a BD Pathway 435 High-content Bioimager (BD Biosciences) or a Zeiss Axiovert 200 fluorescent microscope. A gene was scored as positive only if >20% of animals demonstrated induction. Epistasis analysis was performed by knockdown of each positive regulator as before followed by double RNAi of the positive and negative regulators together.

Each RNAi construct was validated by sequencing. Functional information on the identified genes was collected using WormBase [64].

### Drug Assay

Pharmacological inhibition of the proteasome was conducted using transgenic animals carrying the *phsp70::gfp* reporter grown on standard NGM plates seeded with OP50 bacteria. L4 larval stage animals were incubated with 100 µM MG132 (AG Scientific) in 0.5% DMSO or 0.5% DMSO alone for 2–3 hours and returned to plates. Fluorescence was scored the next day in young adult animals.

### Fluorescence Imaging and Tissue Identification

Transgenic animals carrying the fluorescent reporter were mounted on 3% agarose pads, immobilized with 2 mM levamisole and viewed using the Zeiss Axiovert 200. Animals were imaged using 10X/0.25 A-Plan and 100X/1.4 oil DIC Plan-APOCHROMAT objectives. Images were captured using a Hamamatsu digital camera (C4742-98) with Axiovision Release 4.7 software. Tissue-identification was based on nematode anatomy and tissue morphology using images from the *C. elegans* atlas [65]. A tissue was scored as positive only if >20% of animals demonstrated induction.

### qRT–PCR

RNA was isolated from whole animals lysed by vortexing for twenty minutes after addition of TRIzol (Invitrogen) and DNA was removed using a DNA-free Kit (Ambion) according to standard protocols. cDNA synthesis was performed using an iScript cDNA Synthesis Kit (BioRad) and qRT-PCR was performed using an iQ SYBR Green Supermix Kit (BioRad) using provided protocols and run on a BioRad iCycler. 18S RNA was used as a normalization control.

### Network Analysis

We utilized a graph partitioning scheme that separates the network into groups of nodes, which collectively maximizes the density of within-partition edges in the network [56], [57]. The significance of the number of interactions between the negative regulators was tested by comparing their density to the density of interactions predicted genome-wide in *C. elegans*. The significance of the modularity of the HSR negative regulator network was tested by sampling Monte Carlo realizations in which we exchanged pairs of edges, maintaining the degree distribution of the network.

## Supporting Information

Figure S1 Tissue-selective induction of the *hsp-16.2* reporter (*phsp-16.2::gfp*) by knockdown of negative regulators. Nomarski and fluorescent images corresponding to whole worms and fluorescent images of the muscle tissue, excretory system, and intestine of the *phsp-16.2::gfp* reporter strain are shown. The boundaries of the animals taken from Nomarski images were added as a visual aide to some images. (A–E) In the absence of heat shock, the empty vector control showed no induction above background fluorescence. (F–J) Heat shock induces the reporter in all three tissues. (K–O) RNAi knockdown of *hsp-1* leads to induction of the reporter only in excretory system and intestine; (P–T) knockdown of *daf-21* leads to induction only in muscle; (U–Y) knockdown of *cct-1* leads to induction only in muscle; and (Z–AD) knockdown of *F38A1.8* leads to induction only in the intestine. Images are taken at different exposures to maximize fluorescence of each image. Scale bars of whole animal images correspond to 100 µm, while scale bars of the images depicting specific tissues correspond to 50 µm. Asterisks denote only autofluorescence.
(TIF)

Table S1 Positive and negative regulators of the HSR. Positive and negative regulators of the HSR indentified in the genome-wide screens including cosmid, gene, description, human homologue, percent animals showing induction of *phsp70::gfp* in each tissue, and overlap with screens for polyQ enhancement and suppression.
(PDF)

## References

[1] Akerfelt M, Morimoto RI, Sistonen L (2010) Heat shock factors: integrators of cell stress, development and lifespan. Nat Rev Mol Cell Biol 8: 545–555. 10.1038/nrm2938 PMC3402356 20628411

[2] Craig EA, Gross CA (1991) Is hsp70 the cellular thermometer? Trends Biochem Sci 16: 135–140. 1877088 10.1016/0968-0004(91)90055-z

[3] Abravaya K, Myers MP, Murphy SP, Morimoto RI (1992) The human heat shock protein hsp70 interacts with HSF, the transcription factor that regulates heat shock gene expression. Genes Dev 6: 1153–1164. 1628823 10.1101/gad.6.7.1153

[4] Baler R, Welch WJ, Voellmy R (1992) Heat shock gene regulation by nascent polypeptides and denatured proteins: hsp70 as a potential autoregulatory factor. J Cell Biol 117: 1151–1159. 1607379 10.1083/jcb.117.6.1151 PMC2289502

[5] Mosser DD, Duchaine J, Massie B (1993) The DNA-binding activity of the human heat shock transcription factor is regulated in vivo by hsp70. Mol Cell Biol 13: 5427–5438. 8355691 10.1128/mcb.13.9.5427-5438.1993 PMC360250

[6] Shi Y, Mosser DD, Morimoto RI (1998) Molecular chaperones as HSF1-specific transcriptional repressors. Genes Dev 12: 654–666. 9499401 10.1101/gad.12.5.654 PMC316571

[7] Zou J, Guo Y, Guettouche T, Smith DF, Voellmy R (1998) Repression of heat shock transcription factor HSF1 activation by HSP90 (HSP90 complex) that forms a stress-sensitive complex with HSF1. Cell 94: 471–480. 9727490 10.1016/s0092-8674(00)81588-3

[8] Elefant F, Palter KB (1999) Tissue-specific expression of dominant negative mutant Drosophila HSC70 causes developmental defects and lethality. Mol Biol Cell 10: 2101–2117. 10397752 10.1091/mbc.10.7.2101 PMC25422

[9] Mathur SK, Sistonen L, Brown IR, Murphy SP, Sarge KD, et al (1994) Deficient induction of human hsp70 heat shock gene transcription in Y79 retinoblastoma cells despite activation of heat shock factor 1. Proc Natl Acad Sci U S A 91: 8695–8699. 8078944 10.1073/pnas.91.18.8695 PMC44673

[10] Marcuccilli CJ, Mathur SK, Morimoto RI, Miller RJ (1996) Regulatory differences in the stress response of hippocampal neurons and glial cells after heat shock. J Neurosci 16: 478–485. 8551332 10.1523/JNEUROSCI.16-02-00478.1996 PMC6578639

[11] Batulan Z, Shinder GA, Minotti S, He BP, Doroudchi MM, et al (2003) High threshold for induction of the stress response in motor neurons is associated with failure to activate HSF1. J Neurosci 23: 5789–5798. 12843283 10.1523/JNEUROSCI.23-13-05789.2003 PMC6741252

[12] Blake MJ, Gershon D, Fargnoli J, Holbrook NJ (1990) Discordant expression of heat shock protein mRNAs in tissues of heat-stressed rats. J Biol Chem 265: 15275–15279. 1697588

[13] Morley JF, Morimoto RI (2004) Regulation of longevity in Caenorhabditis elegans by heat shock factor and molecular chaperones. Mol Biol Cell 15: 657–664. 14668486 10.1091/mbc.E03-07-0532 PMC329286

[14] Wu BJ, Williams GT, Morimoto RI (1987) Detection of three protein binding sites in the serum-regulated promoter of the human gene encoding the 70-kDa heat shock protein. Proc Natl Acad Sci U S A 84: 2203–2207. 3031671 10.1073/pnas.84.8.2203 PMC304617

[15] Williams GT, McClanahan TK, Morimoto RI (1989) E1a transactivation of the human HSP70 promoter is mediated through the basal transcriptional complex. Mol Cell Biol 9: 2574–2587. 2474756 10.1128/mcb.9.6.2574-2587.1989 PMC362330

[16] Morgan WD, Williams GT, Morimoto RI, Greene J, Kingston RE, et al (1987) Two transcriptional activators, CCAAT-box-binding transcription factor and heat shock transcription factor, interact with a human hsp70 gene promoter. Mol Cell Biol 7: 1129–1138. 3561411 10.1128/mcb.7.3.1129-1138.1987 PMC365185

[17] Greene JM, Larin Z, Taylor IC, Prentice H, Gwinn KA, et al (1987) Multiple basal elements of a human hsp70 promoter function differently in human and rodent cell lines. Mol Cell Biol 7: 3646–3655. 2824993 10.1128/mcb.7.10.3646-3655.1987 PMC368019

[18] Abravaya K, Phillips B, Morimoto RI (1991) Heat shock-induced interactions of heat shock transcription factor and the human hsp70 promoter examined by in vivo footprinting. Mol Cell Biol 11: 586–592. 1986252 10.1128/mcb.11.1.586-592.1991 PMC359677

[19] Morimoto RI (2008) Proteotoxic stress and inducible chaperone networks in neurodegenerative disease and aging. Genes Dev 22: 1427–1438. 18519635 10.1101/gad.1657108 PMC2732416

[20] Prahlad V, Cornelius T, Morimoto RI (2008) Regulation of the cellular heat shock response in Caenorhabditis elegans by thermosensory neurons. Science 320: 811–814. 18467592 10.1126/science.1156093 PMC3429343

[21] Ben-Zvi A, Miller EA, Morimoto RI (2009) Collapse of proteostasis represents an early molecular event in Caenorhabditis elegans aging. Proc Natl Acad Sci U S A 106: 14914–14919. 19706382 10.1073/pnas.0902882106 PMC2736453

[22] Hsu AL, Murphy CT, Kenyon C (2003) Regulation of aging and age-related disease by DAF-16 and heat-shock factor. Science 300: 1142–1145. 12750521 10.1126/science.1083701

[23] Xiao X, Zuo X, Davis AA, McMillan DR, Curry BB, et al (1999) HSF1 is required for extra-embryonic development, postnatal growth and protection during inflammatory responses in mice. EMBO J 18: 5943–5952. 10545106 10.1093/emboj/18.21.5943 PMC1171660

[24] Jedlicka P, Mortin MA, Wu C (1997) Multiple functions of Drosophila heat shock transcription factor in vivo. EMBO J 16: 2452–2462. 9171358 10.1093/emboj/16.9.2452 PMC1169845

[25] Parker-Thornburg J, Bonner JJ (1987) Mutations that induce the heat shock response of Drosophila. Cell 51: 763–772. 3119225 10.1016/0092-8674(87)90099-7

[26] Bonner JJ, Parks C, Parker-Thornburg J, Mortin MA, Pelham HR (1984) The use of promoter fusions in Drosophila genetics: isolation of mutations affecting the heat shock response. Cell 37: 979–991. 6430570 10.1016/0092-8674(84)90432-x

[27] Silva MC, Fox S, Beam M, Thakkar H, Amaral MD, et al (2011) A genetic screening strategy identifies novel regulators of the proteostasis network. PLoS Genet 7: e1002438 doi:10.1371/journal.pgen.1002438. 22242008 10.1371/journal.pgen.1002438 PMC3248563

[28] Nollen EA, Garcia SM, van Haaften G, Kim S, Chavez A, et al (2004) Genome-wide RNA interference screen identifies previously undescribed regulators of polyglutamine aggregation. Proc Natl Acad Sci U S A 101: 6403–6408. 15084750 10.1073/pnas.0307697101 PMC404057

[29] Shore DE, Carr CE, Ruvkun G (2012) Induction of cytoprotective pathways is central to the extension of lifespan conferred by multiple longevity pathways. PLoS Genet 8: e1002792 doi:10.1371/journal.pgen.1002792. 22829775 10.1371/journal.pgen.1002792 PMC3400582

[30] Melo JA, Ruvkun G (2012) Inactivation of conserved C. elegans genes engages pathogen- and xenobiotic-associated defenses. Cell 149: 452–466. 22500807 10.1016/j.cell.2012.02.050 PMC3613046

[31] Wang MC, Min W, Freudiger CW, Ruvkun G, Xie XS (2011) RNAi screening for fat regulatory genes with SRS microscopy. Nat Methods 8: 135–138. 21240281 10.1038/nmeth.1556 PMC3061290

[32] Curran SP, Ruvkun G (2007) Lifespan regulation by evolutionarily conserved genes essential for viability. PLoS Genet 3: e56 doi:10.1371/journal.pgen.0030056. 17411345 10.1371/journal.pgen.0030056 PMC1847696

[33] Hansen M, Hsu AL, Dillin A, Kenyon C (2005) New genes tied to endocrine, metabolic, and dietary regulation of lifespan from a Caenorhabditis elegans genomic RNAi screen. PLoS Genet 1: e17 doi:10.1371/journal.pgen.0010017. 10.1371/journal.pgen.0010017 PMC1183531 16103914

[34] Wang J, Robida-Stubbs S, Tullet JM, Rual JF, Vidal M, et al (2010) RNAi screening implicates a SKN-1-dependent transcriptional response in stress resistance and longevity deriving from translation inhibition. PLoS Genet 6: e1001048 doi:10.1371/journal.pgen.1001048. 20700440 10.1371/journal.pgen.1001048 PMC2916858

[35] Kamath RS, Fraser AG, Dong Y, Poulin G, Durbin R, et al (2003) Systematic functional analysis of the Caenorhabditis elegans genome using RNAi. Nature 421: 231–237. 12529635 10.1038/nature01278

[36] Kapulkin WJ, Hiester BG, Link CD (2005) Compensatory regulation among ER chaperones in C. elegans. FEBS Lett 579: 3063–3068. 15907843 10.1016/j.febslet.2005.04.062

[37] Calfon M, Zeng H, Urano F, Till JH, Hubbard SR, et al (2002) IRE1 couples endoplasmic reticulum load to secretory capacity by processing the XBP-1 mRNA. Nature 415: 92–96. 11780124 10.1038/415092a

[38] Khaleque MA, Bharti A, Gong J, Gray PJ, Sachdev V, et al (2008) Heat shock factor 1 represses estrogen-dependent transcription through association with MTA1. Oncogene 27: 1886–1893. 17922035 10.1038/sj.onc.1210834

[39] Murawska M, Hassler M, Renkawitz-Pohl R, Ladurner A, Brehm A (2011) Stress-induced PARP activation mediates recruitment of Drosophila Mi-2 to promote heat shock gene expression. PLoS Genet 7: e1002206 doi: 10.1371/journal.pgen.1002206. 21829383 10.1371/journal.pgen.1002206 PMC3145624

[40] Hajdu-Cronin YM, Chen WJ, Sternberg PW (2004) The L-type cyclin CYL-1 and the heat-shock-factor HSF-1 are required for heat-shock-induced protein expression in Caenorhabditis elegans. Genetics 168: 1937–1949. 15611166 10.1534/genetics.104.028423 PMC1448743

[41] Neef DW, Turski ML, Thiele DJ Modulation of heat shock transcription factor 1 as a therapeutic target for small molecule intervention in neurodegenerative disease. PLoS Biol 8: e1000291 doi:10.1371/journal.pbio.1000291. 10.1371/journal.pbio.1000291 PMC2808216 20098725

[42] Guisbert E, Herman C, Lu CZ, Gross CA (2004) A chaperone network controls the heat shock response in E. coli. Genes Dev 18: 2812–2821. 15545634 10.1101/gad.1219204 PMC528900

[43] Poritz MA, Bernstein HD, Strub K, Zopf D, Wilhelm H, et al (1990) An E. coli ribonucleoprotein containing 4.5S RNA resembles mammalian signal recognition particle. Science 250: 1111–1117. 1701272 10.1126/science.1701272

[44] Arnold CE, Wittrup KD (1994) The stress response to loss of signal recognition particle function in Saccharomyces cerevisiae. J Biol Chem 269: 30412–30418. 7982955

[45] Zhou M, Wu X, Ginsberg HN (1996) Evidence that a rapidly turning over protein, normally degraded by proteasomes, regulates hsp72 gene transcription in HepG2 cells. J Biol Chem 271: 24769–24775. 8798747 10.1074/jbc.271.40.24769

[46] Mathew A, Mathur SK, Morimoto RI (1998) Heat shock response and protein degradation: regulation of HSF2 by the ubiquitin-proteasome pathway. Mol Cell Biol 18: 5091–5098. 9710593 10.1128/mcb.18.9.5091 PMC109094

[47] Taubert S, Hansen M, Van Gilst MR, Cooper SB, Yamamoto KR (2008) The Mediator subunit MDT-15 confers metabolic adaptation to ingested material. PLoS Genet 4: e1000021 doi:10.1371/journal.pgen.1000021. 18454197 10.1371/journal.pgen.1000021 PMC2265483

[48] Badenhorst P, Voas M, Rebay I, Wu C (2002) Biological functions of the ISWI chromatin remodeling complex NURF. Genes Dev 16: 3186–3198. 12502740 10.1101/gad.1032202 PMC187504

[49] Kipreos ET (2005) Ubiquitin-mediated pathways in C. elegans. WormBook 1–24. 10.1895/wormbook.1.36.1 PMC4781597 18050424

[50] Anderson LL, Mao X, Scott BA, Crowder CM (2009) Survival from hypoxia in C. elegans by inactivation of aminoacyl-tRNA synthetases. Science 323: 630–633. 19179530 10.1126/science.1166175 PMC3739282

[51] Segref A, Torres S, Hoppe T (2011) A screenable in vivo assay to study proteostasis networks in Caenorhabditis elegans. Genetics 187: 1235–1240. 21288877 10.1534/genetics.111.126797 PMC3070531

[52] Link CD, Cypser JR, Johnson CJ, Johnson TE (1999) Direct observation of stress response in Caenorhabditis elegans using a reporter transgene. Cell Stress Chaperones 4: 235–242. 10590837 10.1379/1466-1268(1999)004<0235:doosri>2.3.co;2 PMC312938

[53] Murphy CT, McCarroll SA, Bargmann CI, Fraser A, Kamath RS, et al (2003) Genes that act downstream of DAF-16 to influence the lifespan of Caenorhabditis elegans. Nature 424: 277–283. 12845331 10.1038/nature01789

[54] Caruso ME, Jenna S, Bouchecareilh M, Baillie DL, Boismenu D, et al (2008) GTPase-mediated regulation of the unfolded protein response in Caenorhabditis elegans is dependent on the AAA+ ATPase CDC-48. Mol Cell Biol 28: 4261–4274. 18458060 10.1128/MCB.02252-07 PMC2447140

[55] Zhong W, Sternberg PW (2006) Genome-wide prediction of C. elegans genetic interactions. Science 311: 1481–1484. 16527984 10.1126/science.1123287

[56] Newman ME, Girvan M (2004) Finding and evaluating community structure in networks. Phys Rev E Stat Nonlin Soft Matter Phys 69: 026113. 14995526 10.1103/PhysRevE.69.026113

[57] Guimera R, Amaral LA (2005) Cartography of complex networks: modules and universal roles. J Stat Mech 2005: nihpa35573. 18159217 10.1088/1742-5468/2005/02/P02001 PMC2151742

[58] Yoneda T, Benedetti C, Urano F, Clark SG, Harding HP, et al (2004) Compartment-specific perturbation of protein handling activates genes encoding mitochondrial chaperones. J Cell Sci 117: 4055–4066. 15280428 10.1242/jcs.01275

[59] Ron D, Walter P (2007) Signal integration in the endoplasmic reticulum unfolded protein response. Nat Rev Mol Cell Biol 8: 519–529. 17565364 10.1038/nrm2199

[60] Reddy PS, Corley RB (1999) The contribution of ER quality control to the biologic functions of secretory IgM. Immunol Today 20: 582–588. 10562710 10.1016/s0167-5699(99)01542-x

[61] Dunn AY, Melville MW, Frydman J (2001) Review: cellular substrates of the eukaryotic chaperonin TRiC/CCT. J Struct Biol 135: 176–184. 11580267 10.1006/jsbi.2001.4380

[62] Brenner S (1974) The genetics of Caenorhabditis elegans. Genetics 77: 71–94. 4366476 10.1093/genetics/77.1.71 PMC1213120

[63] McKay SJ, Johnsen R, Khattra J, Asano J, Baillie DL, et al (2003) Gene expression profiling of cells, tissues, and developmental stages of the nematode C. elegans. Cold Spring Harb Symp Quant Biol 68: 159–169. 15338614 10.1101/sqb.2003.68.159

[64] Harris TW, Antoshechkin I, Bieri T, Blasiar D, Chan J, et al WormBase: a comprehensive resource for nematode research. Nucleic Acids Res 38: D463–467. 19910365 10.1093/nar/gkp952 PMC2808986

[65] Hall DH, Altun ZF (2008) C. elegans atlas. Cold Spring Harbor, N.Y.: Cold Spring Harbor Laboratory Press. x, 348 p. p.

