# Supplementary figures and images for: Identification of a Tissue-Selective Heat Shock Response Regulatory Network
Source: PLoS Genet. 2013 Apr 18;9(4):e1003466. doi: 10.1371/journal.pgen.1003466 (PMC3630107; doi:10.1371/journal.pgen.1003466)

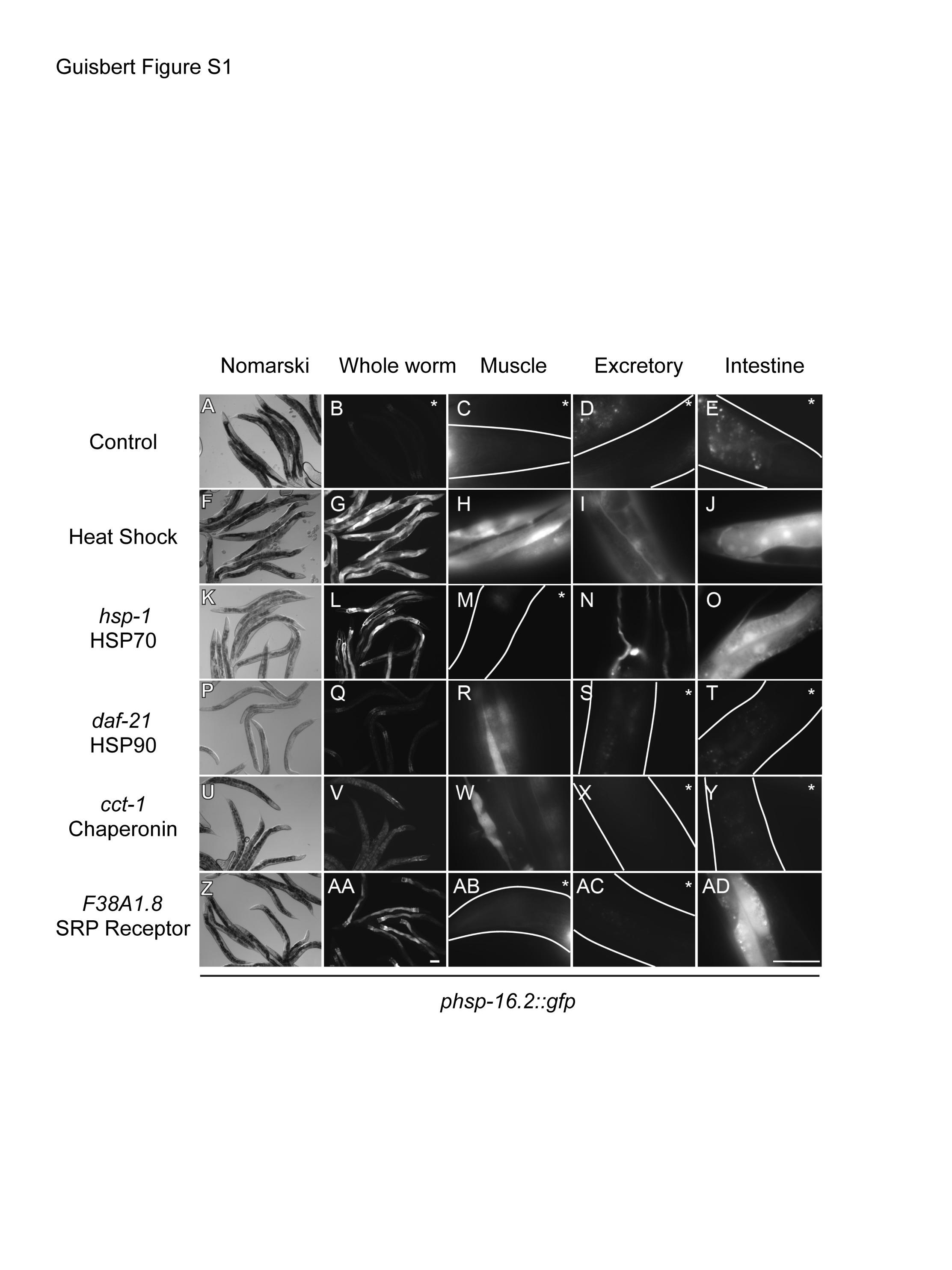

Supplement: Figure S1 — Tissue-selective induction of the hsp-16.2 reporter (phsp-16.2::gfp) by knockdown of negative regulators. Nomarski and fluorescent images corresponding to whole worms and fluorescent images of the muscle tissue, excretory system, and intestine of the phsp-16.2::gfp reporter strain are shown. The boundaries of the animals taken from Nomarski images were added as a visual aide to some images. (A–E) In the absence of heat shock, the empty vector control showed no induction above background fluorescence. (F–J) Heat shock induces the reporter in all three tissues. (K–O) RNAi knockdown of hsp-1 leads to induction of the reporter only in excretory system and intestine; (P–T) knockdown of daf-21 leads to induction only in muscle; (U–Y) knockdown of cct-1 leads to induction only in muscle; and (Z–AD) knockdown of F38A1.8 leads to induction only in the intestine. Images are taken at different exposures to maximize fluorescence of each image. Scale bars of whole animal images correspond to 100 µm, while scale bars of the images depicting specific tissues correspond to 50 µm. Asterisks denote only autofluorescence. (TIF) [file pgen.1003466.s001.tif]
