# Supplementary material for: Identification of a Tissue-Selective Heat Shock Response Regulatory Network
Source: PLoS Genet. 2013 Apr 18;9(4):e1003466. doi: 10.1371/journal.pgen.1003466 (PMC3630107; doi:10.1371/journal.pgen.1003466)
Supplement: Table S1 — Positive and negative regulators of the HSR. Positive and negative regulators of the HSR indentified in the genome-wide screens including cosmid, gene, description, human homologue, percent animals showing induction of phsp70::gfp in each tissue, and overlap with screens for polyQ enhancement and suppression. (PDF) [file pgen.1003466.s002.pdf]

Supplemental Table 1: Positive and Negative HSR Regulators

Cosmid	Gene	Description	Human	*hsp70::gfp* Induction (% +/- SEM)				Overlap
				S	I	B	V	
F26D10.3	*hsp-1*	HSP70 Chaperone	HSPA8	100 +/- 0	100 +/- 0	97 +/- 3	100 +/- 0	N, L
C47E8.5	*daf-21*	HSP90 Chaperone	HSP90AB1	69 +/- 16	56 +/- 16	87 +/- 6	81 +/- 11	
T05C12.7	*cct-1*	CCT/TRiC Chaperone	TCP1			100 +/- 0	95 +/- 5	N
T21B10.7	*cct-2*	CCT/TRiC Chaperone	CCT2			95 +/- 5	88 +/- 6	N, L
F54A3.3	*cct-3*	CCT/TRiC Chaperone	CCT3			94 +/- 6	83 +/- 11	
K01C8.10	*cct-4*	CCT/TRiC Chaperone	CCT4			94 +/- 6	98 +/- 2	N, L
C07G2.3	*cct-5*	CCT/TRiC Chaperone	CCT5			100 +/- 0	100 +/- 0	N
F01F1.8	*cct-6*	CCT/TRiC Chaperone	CCT6A			93 +/- 7	90 +/- 6	N
T10B5.5	*cct-7*	CCT/TRiC Chaperone	CCT7			80 +/- 13	92 +/- 8	N, L
Y55F3AR.3	*cct-8*	CCT/TRiC Chaperone	CCT8			100 +/- 0	100 +/- 0	L
R05F9.10	*sgt-1*	TPR cochaperone	SGTA			20 +/- 20	25 +/- 25	
F30H5.1	*unc-45*	TPR cochaperone	UNC45B			53 +/- 5	98 +/- 3	
T01B7.4	*cyn-11*	Cyclophilin cochaperone	PPIH			22 +/- 7		
C36B1.4	*pas-4*	Proteasome 20S subunit	PSMA7	100 +/- 0	87 +/- 11			N, L
F25H2.9	*pas-5*	Proteasome 20S subunit	PSMA5	98 +/- 2	92 +/- 6			
CD4.6	*pas-6*	Proteasome 20S subunit	PSMA1	100 +/- 0	82 +/- 13			N, L
C47B2.4	*pbs-2*	Proteasome 20S subunit	PSMB7	80 +/- 10	84 +/- 8			N
Y38A8.2	*pbs-3*	Proteasome 20S subunit	PSMB3	100 +/- 0	100 +/- 0			N
T20F5.2	*pbs-4*	Proteasome 20S subunit	PSMB2	100 +/- 0	93 +/- 7			N
K05C4.1	*pbs-5*	Proteasome 20S subunit	PSMB8	92 +/- 8	97 +/- 3			N
C02F5.9	*pbs-6*	Proteasome 20S subunit	PSMB1	100 +/- 0	97 +/- 3			
F39H11.5	*pbs-7*	Proteasome 20S subunit	PSMB4	100 +/- 0	93 +/- 5			
C52E4.4	*rpt-1*	Proteasome 19S subunit	PSMC2	82 +/- 14	70 +/- 11			
F23F12.6	*rpt-3*	Proteasome 19S subunit	PSMC4	86 +/- 7	79 +/- 10			
F23F1.8	*rpt-4*	Proteasome 19S subunit	PSMC6	54 +/- 21	65 +/- 16			
F56H1.4	*rpt-5*	Proteasome 19S subunit	PSMC3	47 +/- 13	63 +/- 13			N
Y49E10.1	*rpt-6*	Proteasome 19S subunit	PSMC5	91 +/- 7	94 +/- 4			
T22D1.9	*rpn-1*	Proteasome 19S subunit	PSMD2	73 +/- 16	91 +/- 6			L
C23G10.4	*rpn-2*	Proteasome 19S subunit	PSMD1	88 +/- 12	77 +/- 13			L
F57B9.10	*rpn-6*	Proteasome 19S subunit	PSMD11	66 +/- 12	64 +/- 6			N, L
F49C12.8	*rpn-7*	Proteasome 19S subunit	PSMD6	66 +/- 13	70 +/- 10			
R12E2.3	*rpn-8*	Proteasome 19S subunit	PSMD7	92 +/- 4	86 +/- 7			N
K07D4.3	*rpn-11*	Proteasome 19S subunit	PSMD14	73 +/- 15	69 +/- 15			N
F57B10.1	*let-607*	CREB/ATF TF (ER)	CREB3L3	33 +/- 19	68 +/- 19			
C15H9.6	*hsp-3*	HSP70 Chaperone (ER)	HSPA5	82 +/- 7	78 +/- 10			
F38A1.8		SRP recepter α	SRPR	94 +/- 4	100 +/- 0			
R186.3		SRP recepter β	SRPRB	23 +/- 10	26 +/- 12			
F55C5.8		SRP68	SRP68	85 +/- 7	100 +/- 0			L
F08D12.1		SRP72	SRP72		70 +/- 9			
F25G6.8		SRP14	SRP14P1		24 +/- 12			

F38E11.5		COP1 β' subunit	COPB2		55 +/- 26			L
T14G10.5		COP1 γ subunit	COPG2		27 +/- 17			
T24H7.2		HSP70 Chaperone (ER)	HYOU1		24 +/- 12			
C37H5.8	*hsp-6*	HSP70 Chaperone (mito)	HSPA9B		81 +/- 12			N
T09B4.9		TIM44 mitochondial import	TIMM44		23 +/- 16			
C47E12.5	*uba-1*	E1 ubiquitin ligase	UBA1		31 +/- 14			N
F52C6.3	*phi-32*	Ubiquitin	UBC		32 +/- 20			N
C53A5.6		E3 ubiquitin ligase	IPP		40 +/- 6			
B0464.1	*drs-1*	Asp tRNA Synthetase	DARS		99 +/- 1			
W04A4.5		Integrator subunit	INTS4		30 +/- 14			
C47E12.4	*pyp-1*	NuRF subunit	PPA1		63 +/- 20			
R12B2.5	*mdt-15*	Mediator subunit	MED15		26 +/- 12			
C26C6.5	*dcp-66*	NuRD subunit	GATAD2B	N/A	N/A	N/A	N/A	
F09D1.1		USP39	USP39	N/A	N/A	N/A	N/A	
T28D9.10	*snr-3*	snRNP D1	SNRPD1	N/A	N/A	N/A	N/A	N, L
T08A11.2	*phi-11*	Splicing factor 3b, subunit 1	SF3B1	N/A	N/A	N/A	N/A	N, L
ZK328.2	*eftu-2*	EF-2 like	EFTUD2	N/A	N/A	N/A	N/A	N
T13H5.4	*phi-8*	Splicing factor 3a, subunit 3	SF3A3	N/A	N/A	N/A	N/A	N, L
Y53C10A.12	*hsf-1*	heat shock factor	HSF1	N/A	N/A	N/A	N/A	N, L

S = Spermatheca, I = Intestine, B = Body wall muscle, V = Vulva muscle, N = Nollen *et al.,* L = Lamitina *et al.*
